# Shared Decision-Making for a Dialysis Modality

**DOI:** 10.1016/j.ekir.2021.10.019

**Published:** 2021-10-30

**Authors:** Xueqing Yu, Masaaki Nakayama, Mai-Szu Wu, Yong-Lim Kim, Lily Mushahar, Cheuk Chun Szeto, Dori Schatell, Fredric O. Finkelstein, Robert R. Quinn, Michelle Duddington

**Affiliations:** 1 Division of Nephrology, Guangdong Provincial People’s Hospital, Guangzhou, People’s Republic of China; 2 Kidney Center, St. Luke’s International Hospital, Tokyo, Japan; 3 Division of Nephrology, Department of Internal Medicine, School of Medicine, College of Medicine, Taipei Medical University, Taipei, Taiwan; 4 Division of Nephrology, Department of Internal Medicine, Taipei Medical University-Shuang Ho Hospital, New Taipei City, Taiwan; 5 Department of Internal Medicine, School of Medicine, Kyungpook National University, Daegu, Republic of Korea; 6 Department of Nephrology, Hospital Tuanku Ja'afar, Seremban, Malaysia; 7 Department of Medicine & Therapeutics, Faculty of Medicine, The Chinese University of Hong Kong, Shatin, Hong Kong; 8 Medical Education Institute, Inc., Madison, Wisconsin, USA; 9 Yale School of Medicine, New Haven, Connecticut, USA; 10 Department of Community Health Sciences, Cumming School of Medicine, University of Calgary, Calgary, Alberta, Canada; 11 Baxter Healthcare, Sydney, New South Wales, Australia

**Keywords:** continuous renal replacement therapy, education, hemodialysis, peritoneal dialysis

## Abstract

The prevalence of kidney failure continues to rise globally. Dialysis is a treatment option for individuals with kidney failure; after the decision to initiate dialysis has been made, it is critical to involve individuals in the decision on which dialysis modality to choose. This review, based on evidence arising from the literature, examines the role of shared decision-making (SDM) in helping those with kidney failure to select a dialysis modality. SDM was found to lead to more people with kidney failure feeling satisfied with their choice of dialysis modality. Individuals with kidney failure must be cognizant that SDM is an active and iterative process, and their participation is essential for success in empowering them to make decisions on dialysis modality. The educational components of SDM must be easy to understand, high quality, unbiased, up to date, and targeted to the linguistic, educational, and cultural needs of the individual. All individuals with kidney failure should be encouraged to participate in SDM and should be involved in the design and implementation of SDM approaches.

Chronic kidney disease (CKD) is defined as kidney damage or an estimated glomerular filtration rate <60 ml/min per 1.73 m^2^ for ≥3 months, regardless of cause.^1^ Individuals with CKD can develop end-stage kidney disease, also known as kidney failure, which is defined as an estimated glomerular filtration rate <15 ml/min per 1.73 m^2^.^1^ In 2017, the estimated global prevalence of CKD was 9.1%, with 0.04% of the population receiving dialysis.^2^ The prevalence of kidney failure continues to rise worldwide and up to 5 million people are expected to require treatment by 2030, with the greatest increase in Asia.^3^^,^^4^ Equity of access to kidney replacement therapy varies between countries based on finance and rationing.^5^ Where available, kidney transplantation is the preferred option in eligible individuals.^6^ Dialysis options for individuals with kidney failure include hemodialysis (HD), which can either be undertaken in-center (ICHD) or at home (HHD), or peritoneal dialysis (PD), which can be continuous ambulatory PD or automated PD. Evidence suggests that individuals with kidney failure receiving PD treatment have a similar risk of death to those receiving ICHD.^4^^,^^7^, ^8^, ^9^, ^10^, ^11^, ^12^, ^13^, ^14^, ^15^, ^16^

### Helping the Individual Make a Treatment Choice

There are different treatment decision-making models to aid individuals seeking health care.^17^

1. In the parental or paternalistic model, the health care professional (HCP) decides on the treatment strategy.
2. The HCP as best agent model also focuses on treatment options being decided by the HCP but considers the values and preferences of the individual seeking health care.
3. The shared decision-making (SDM) model allows for open discussion between the individual seeking health care and the HCP and uses a collaborative approach to choose a treatment.
4. Informed decision-making allows the individual seeking health care to make a decision on their treatment based on information supplied to them by the HCP without collaboration on the treatment decision (an “informed choice”).

Of course, people have the intrinsic right to refuse treatment.^18^ Although individual rights may vary by country or jurisdiction, the clinician has a duty of care to respect the rights of the individual seeking health care.^18^ Furthermore, patient involvement is increasingly recognized as essential in research, quality improvement, policy development, service reviews, and payer reviews.^19^^,^^20^ As such, individuals seeking health care should be more involved in decisions on their own treatment and care. Indeed, the phrase “no decision about me, without me” has become synonymous with SDM.^21^

SDM provides individuals seeking health care with the opportunity for engagement and has been defined as an “approach where clinicians and patients make decisions together using the best available evidence … Shared decision making respects patient autonomy and promotes patient engagement.”^22^ It has also been found that SDM can lead to increased satisfaction, reduce anxiety, improve treatment compliance, and lower demand for health care resources.^23^, ^24^, ^25^, ^26^, ^27^, ^28^

A randomized trial has found the importance of choice to individuals with kidney failure on to start dialysis. This trial, which compared starting therapy with ICHD versus PD, was stopped early because of difficulties with recruitment when only 38 of more than 700 eligible participants agreed to be randomized. The authors suggested that one possible explanation for the vast majority of eligible individuals with kidney failure refusing consent to participate in the trial was that they had already developed a treatment preference after receiving extensive education on the available options and were unwilling to be randomized to their nonpreferred choice.^29^ Following predialysis education, it has been suggested that up to 50% of informed individuals would choose home dialysis options,^30^ yet the reality remains that individuals with kidney failure receiving PD and HHD represent a small proportion of the global dialysis population.^4^^,^^31^^,^^32^

Systematic reviews of qualitative studies have revealed that HHD and PD improve an individual with kidney failure’s sense of independence, self-efficacy, and well-being, but they may also experience anxiety owing to isolation from medical and social support and need strategies to help improve resilience and self-confidence.^33^^,^^34^

This review, based on expert opinions and evidence arising from published literature, describes the processes that enable effective SDM, illustrates how SDM is used for predialysis education, identifies the barriers to the uptake of SDM, and provides suggestions on how to overcome these barriers. For literature regarding SDM and dialysis modality choice to be included in this narrative review, we performed a PubMed search using the terms “chronic kidney disease,” “shared decision-making,” and “dialysis.” Literature in English published after 2000 was evaluated, and all authors contributed to inclusion of articles and proposed additional literature based on expert opinion and experience in the area. Articles were screened by title, followed by abstract, and then by full text. Literature detailing treatment options for kidney failure other than dialysis was excluded.

### The SDM Model

A number of resources from Healthwise (USA),^35^ the United Kingdom National Health Service,^36^ and the SHARE approach from the US Agency for Healthcare Research and Quality^37^ have outlined the core steps associated with SDM, including the following:

1. Inviting the individual seeking health care to participate in the SDM process.
2. Allowing information exchange between the person seeking health care and the HCP, supplemented with evidence-based resources, such as patient decision aids (PDAs).
3. An assessment of the individual’s values and preferences to reach a common understanding on risks and benefits of the treatment options.
4. Enabling the individual seeking health care to arrive at an initial/preferred treatment decision, which is then discussed with the HCP.
5. Attaining and reviewing a final decision.

An ideal consultation leading to the choice of a dialysis modality would involve building a trusting relationship between the HCP and individual with kidney failure and attending to any care-associated emotional needs of the individual. Clinical findings should be discussed at a suitable health literacy level. This could result in an agreement on a unique dialysis management plan that respects the preferences of the individual with kidney failure and incorporates both quality- and quantity-of-life considerations.

To help HCPs integrate SDM into their practice, a 3-step model, developed from a sample of the general population, has been proposed to guide consultations with individuals seeking health care (Figure 1). This model uses “Choice,” “Option,” and “Decision” talks, during which participants are provided with decision support materials to assist them in making their treatment decisions using a deliberative process.^38^

**Figure 1:**
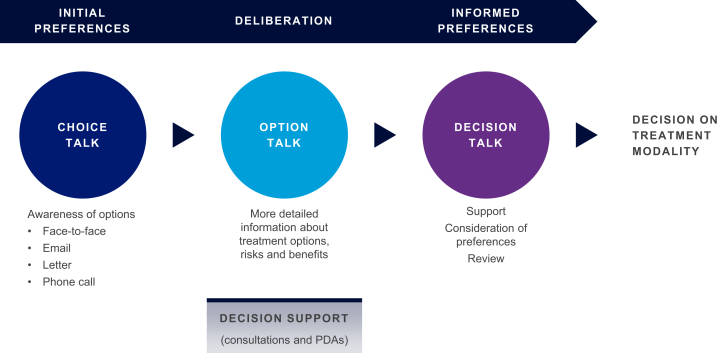
The 3-talk shared decision-making model. Adapted from Elwyn G, Frosch D, Thomson R, et al. Shared decision making: a model for clinical practice. *J Gen Intern Med.* 2012;27:1361–1367.^38^ © The Author(s) 2012. This article is published as open access at https://link.springer.com/article/10.1007%2Fs11606-012-2077-6. PDA, patient decision aid.

### The Role of PDAs in SDM and Predialysis Education

PDAs help individuals seeking health care arrive at informed choices on screening, treatment, or other interventions^39^; they are not designed to replace consultations with the HCP. PDAs can be delivered in a variety of formats, including booklets, interactive media, and video or audio sessions.^39^ It is important that the content of PDAs be evidence based, devoid of bias, and verifiable because of the influence the tool may have on decision-making.^39^ To support confidence in the quality of the PDA for both HCPs and individuals seeking health care, the International Patient Decision Aids Standards Collaboration developed a certification tool^40^ and a set of criteria for quality appraisal of PDAs. The key criteria were “content” (key information, probabilities, clarification of values, and disease-specific guidance), “development process” (generic design and developmental criteria for PDAs), and “effectiveness” (generic evidence of high-quality decision processes and a high-quality choice).^39^

A Cochrane review of PDAs for individuals seeking health care facing screening decisions reported that those who used a PDA, versus those who did not, felt they were better informed and had a more active role when choosing their treatments. Indeed, there was an increase in knowledge and understanding of risk perception and a decrease in decisional conflict and the number of individuals who were undecided on their treatment choices. Importantly, PDAs were also found to improve communication between individuals seeking health care and HCPs and improve satisfaction in their treatment decisions.^41^ Table 1 provides examples of PDAs that have been developed for individuals with kidney failure receiving or on to start dialysis treatment.

**Table 1:** Examples of PDAs used for individuals with kidney failure receiving or on to start dialysis treatment

Name	Country	Format^a^	Target	Link/reference
My Kidneys My Choice	Australia, NZ	Online, PDF, paper	Individuals who will experience kidney failure in 6–12 mo	https://kidney.org.au/your-kidneys/treatment/my-kidneys-my-choice
The Dialysis Decision Aid Booklet: Making the Right Choices For You	UK	PDF, paper	Individuals with CKD or their carers	https://kidneyresearchuk.org/wp-content/uploads/2019/05/KR-decision-Aid-DOWNLOAD.pdf
Ontario Renal Network SHERPA PDA	Canada	PDF	Individuals with kidney failure who would like to plan which dialysis treatment option is best for them and/or want to share their views with others	https://www.ontariorenalnetwork.ca/en/kidney-care-resources/clinical-tools/person-centred-care
Healthwise PDAs: 1. Kidney Failure: What Type of Dialysis Should I Have? 2. Kidney Failure: When Should I Start Dialysis? 3. Advance Care Planning: Should I Stop Kidney Dialysis? 4. Kidney Failure: Should I Start Dialysis?	USA	Online	Individuals with the following: 1. Kidney failure considering dialysis methods 2. Kidney failure who have decided on dialysis but are uncertain when to start 3. Kidney failure treated with dialysis, who are considering stoppage of treatment 4. Kidney failure considering dialysis	https://www.healthwise.org/services.aspx
My Life, My Dialysis Choice	USA	Online	Individuals who have the following: 1. Late-stage CKD and need to make a choice 2. Urgent dialysis start and no previous education 3. A failed transplant 4. A current modality they are unhappy with	https://mydialysischoice.org/
Preparing For Kidney Treatment: You Have a Choice	USA	Paper and video	Individuals with kidney failure receiving dialysis or yet to start treatment	Ameling *et al.*^42^
iChoose Kidney	USA	Online	Individuals with kidney failure deciding on dialysis options vs. transplant	https://ichoosekidney.emory.edu/

CKD, chronic kidney disease; NZ, New Zealand; PDA, patient decision aid; UK, United Kingdom; USA, United States of America.

a Online: interactive online platform; PDF: online digital material; paper: physical print (e.g., brochure); video: online video format.

A total of 17 PDAs designed to support individuals with kidney failure choose between dialysis and conservative management were identified in a systematic review by Winterbottom *et al.*^43^ Of these 17 PDAs, 8 focused on the choice of dialysis modality.

Evaluation of the identified PDAs found variations in the quality of information provided on dialysis modality choice. *The Dialysis Decision Aid Booklet: Making the Right Choices For You* highlighted in Table 1 scored highly in quality assessments; however, none of the PDAs identified included information on treatment failure or switching. They also found variations in how dialysis modality options were described.^43^

The Yorkshire Dialysis Decision Aid (YoDDA) is distributed in *The Dialysis Decision Aid Booklet*. This evidence-based booklet was developed for individuals with kidney failure and their families to facilitate making an informed choice between either HD (ICHD or HHD) or PD (automated PD or continuous ambulatory PD). The decision aid was developed using rigorous decision analysis and behavioral decision support guidance methodology. Clinical guidelines, surveys of dialysis choice, and existing patient information were reviewed as part of the development process.^44^ Content included information on CKD and progressive kidney disease, kidney failure, dialysis options, decision aids focusing on lifestyle activities, and treatment preferences. A study comparing usual care with or without the Yorkshire Dialysis Decision Aid booklet found that individuals with kidney failure who received the Yorkshire Dialysis Decision Aid booklet had higher scores for understanding kidney disease, understanding advantages and disadvantages regarding treatment options, feeling in control over dialysis choice, and sharing their decision with their family compared with those who did not receive the booklet. Individuals deciding on a treatment option considered the views of their family and HCPs as important in decision-making, but the views of others with kidney failure were not rated as important.^44^

In a Spanish registry study, individuals with kidney failure who participated in an education process (4 phases: identifying values, provision of balanced information, deliberation/question and answer, decision-making) with PDAs chose PD significantly more often than those who did not participate (47.8% vs. 6.5%, respectively; *P* < 0.001). Moreover, the use of PDAs, even in the scenario of an unplanned start for dialysis, led to high levels of agreement between the modality of choice and the actual modality that was started in those who had participated in the education process. This suggests that individuals with kidney failure who undertook the education process were more confident in their modality choice.^45^

### Challenges With PDAs

There are numerous challenges regarding the use of PDAs. Clear guidance is needed on who is responsible for updating these tools and how frequently updates are made. It is critical that the information contained within PDAs is evidence based and current as poor-quality, time-consuming, inaccurate, imbalanced, or misleading tools can be harmful to individuals seeking health care.^46^ Clear pathways for funding and certification of PDAs are also lacking.^40^^,^^46^

Another challenge with PDAs is the changing values individuals with kidney failure may have owing to the worsening of their disease. A PDA that is suitable at the beginning of their disease may not be suitable a few years further on, with treatment plans needing to be updated in line with the person’s current situation and values.^43^

Individuals with kidney failure may have cognitive changes that can impair mental skills such as executive function and memory. It is vital to consider the timing of PDAs so the individual with kidney failure can participate in SDM before their decision-making ability has declined.^47^

The challenges with PDAs have been highlighted by The Dialysis Guide, an application for mobile devices developed for a Danish cross-sectional study to help individuals with kidney failure make decisions on dialysis modality. The application included information on kidney failure, dialysis choice, and dialysis modalities, and, although it seemed to help predialysis individuals with kidney failure decide on a dialysis modality, it did not reduce decisional conflict.^48^ The lack of effect on decisional conflict may be because of the fact that 91% of individuals with kidney failure had already attended education sessions or a consultation with an HCP to discuss dialysis options. The usability of the application was rated as low by the participants, whose average age was 65 years. This highlights the need to consider the appropriate timing of PDA deployment when individuals are choosing a dialysis modality, with the educational needs of the individual factored into the material.

### SDM for Predialysis Individuals With Kidney Failure

Individuals with kidney failure who are yet to start dialysis are often not given enough information to make an informed choice or have their choices made for them. Indeed, almost one-third of participants (32.2%) interviewed in the Empowering Patients On Choices for Renal Replacement Therapy (EPOCH-RRT) study, of whom 46.8% started ICHD and only 2.6% started PD, did not think the decision to start dialysis was primarily their choice.^49^ These data suggest that individuals who received limited information and opportunity to make an informed choice were unlikely to choose PD as a dialysis modality.

Shared Decision Making and Dialysis Choice (SDM-DC) program is an initiative received by 348 individuals with kidney failure at 4 hospitals in Denmark who were making decisions on starting HD or PD. The intervention was based on the “three-talk model”^38^ and included a PDA, videos, and 3 meetings between individuals with kidney failure and a dialysis coordinator. As a result of partaking in the Shared Decision Making and Dialysis Choice program, participants felt that they were in control of their dialysis choice. They reported that the meetings were critical to their decision-making and provided them with time and opportunities to ask questions. The PDA was also found to be essential, with individuals in the study reporting that they could not have made a decision without the tool.^50^^,^^51^ A mixed-methods (questionnaire and semistructured interviews) descriptive study of the Shared Decision Making and Dialysis Choice program found that more than 80% of individuals in the trial experienced SDM and made a “high-quality” decision (based on chosen dialysis modality and knowledge scores). The decision quality was the same for those who chose HD or PD.^52^

Of interest is an SDM program conducted in Taiwan that included physician training and a PDA, interviews with individuals with kidney failure, and clinical consultations. Physicians attended a short course that introduced SDM and gave guidance on how to inform and encourage their patients to participate in SDM. Individuals with kidney failure who participated in the program were more likely to choose PD, had higher confidence in their choices for treatment, and experienced less decisional conflict.^53^

The International Society for Peritoneal Dialysis has released key practice recommendations that include SDM: “PD should be prescribed using SDM between the person doing PD and the care team. The aim is to establish realistic care goals that (1) maintain quality of life for the person doing PD as much as possible by enabling them to meet their life goals, (2) minimize symptoms and treatment burden while (3) ensuring high-quality care is provided” (key recommendation 1) and “The PD prescription should take into account the local country resources, the wishes and lifestyle considerations of people needing treatment, including those of their families/caregivers’, especially if providing assistance in their care” (key recommendation 2).^54^

### Barriers to the Implementation of SDM and How to Overcome Them

#### Emotional Burden Experienced by Individuals With Kidney Failure

Individuals with kidney failure may be fearful or overwhelmed when making a decision on initiating dialysis and which modality to choose, and this emotional overload may make it impossible to learn.^55^ Improving feelings of hope can help combat these negative emotions and has been found to significantly reduce fear, anxiety, and depression.^56^, ^57^, ^58^ An 8-week stress management intervention provided to individuals having ICHD by HD nurses was found to significantly increase hope compared with those who did not receive the intervention (*P* < 0.001).^57^ Hope was also found to be an independent predictor for psychological adjustment of individuals with kidney failure on dialysis and was inversely associated with anxiety and depression and positively associated with mental health quality of life.^58^ The integration of interventions to promote hope within the SDM process could lead to better engagement and outcomes for individuals with kidney failure, who in turn may act as patient ambassadors involved in the SDM process, alleviating feelings of fear and anxiety in other individuals with kidney failure.

#### Psychosocial Factors and Willingness to Engage in SDM

Despite having a strong desire for more information, individuals with kidney failure are not always willing to participate in SDM, especially those who have low ratings for autonomy, are older, male, or of non-White race.^59^ Studies have found that males were more likely to delay making a decision on a dialysis modality than females, with females more willing to seek information regarding dialysis modalities.^60^ Ethnic minorities face a number of barriers to access SDM, one of which may be a mistrust in medical professionals.^61^ Involving the individual’s spouse, family, and community can help with informal education of the SDM participant and identification of factors that influence preferences for engagement may help the provision of individually tailored care.

Importantly, the emotional, psychosocial, and cognitive states of the individual with kidney failure when considering starting dialysis have an impact on the choices made. Anxiety is highly prevalent among individuals with kidney failure undergoing dialysis and is associated with an increase in the 1-year hospitalization rate, length of hospital stay, and all-cause mortality.^62^ Individuals with kidney failure were found to be less likely to regret their choice to start dialysis when they had discussions with their HCP on life expectancy and were more likely to regret their decision if they chose to have dialysis to please family members or their doctor.^63^ As expected, treatment satisfaction was higher in those who had participated in SDM and had a “good” psychological state (less anxious and/or depressed) when starting treatment.^64^

### Lack of HCP Engagement

Individuals with kidney failure are not the only ones who may not engage in SDM^59^; HCPs have reported that the challenges to implementing SDM include the lack of available tools and training, their patients not wanting to participate in SDM, and high workload.^65^, ^66^, ^67^, ^68^ Time constraints may be resolved by asking patients to work through a tool before the visit with the HCP or for another member of the multidisciplinary team to engage with the individual, in addition to the physician.

The medical community must ensure young and emerging nephrologists are trained and confident with SDM. Unless they are fully equipped with the information to make decisions, individuals with kidney failure will continue to miss out on opportunities to participate in their own care. It is also important to identify whether education tools need to be developed or if existing tools are of an adequate standard and the barrier to effective SDM is access to resources. Moreover, it is necessary that the nephrology community ensures they have adequate knowledge and understanding of new and emerging evidence—presenting outdated and biased evidence is no longer acceptable. The modern era has seen advances in outcome data, such as technique survival and patient survival, demonstrating, for example, that PD is no longer inferior to HD.^4^^,^^7^, ^16^^,^^69^^,^^70^ HCPs need to be trained in a variety of aspects of SDM, including support for the individual with kidney failure, identification of cultural barriers, communication strategies, and recognizing psychosocial factors, such as anxiety. However, a general review of SDM training programs for HCPs identified that many are not evaluated—a major barrier to the improvement of this important aspect of SDM.^71^

HCPs have been found to be poor at evaluating their patients’ preferred treatment and level of involvement in decision-making, even in institutions that have implemented SDM.^72^ It has been found that “paternalist” and “institutionalist” HCPs often perceive the initiation of dialysis for older individuals as a success, a view often at odds with the perception of the individual themselves. HCPs who engaged in SDM with their patients focused more on quality of life and aligning values with treatment and were the only ones who offered conservative care to those of an older age.^73^ It is important that the values and preferences of the HCP are not transferred to the individual seeking health care as the preferred option and that the choice of modality for dialysis is based on the unbiased information provided. Nevertheless, it must be acknowledged that HCPs are bound by local policy. In countries with a PD-first policy, the treatment options that HCPs can offer may be limited.

HCPs may be unwilling to participate in SDM if it is not initiated by the individual seeking health care. In a study of physician interactions with their patients (primary care patients, individuals with systemic lupus erythematosus, or individuals with lung cancer), it was reported that 84% of SDM-like behaviors in medical consultations were not initiated by the HCP.^74^ Individuals seeking health care who engaged in active participation were more likely to receive facilitative communication from the HCP, be educated, and be of White race.^74^ Another study in individuals with depression found that HCPs only initiated SDM after specific requests regarding treatment were made.^75^ It has been suggested that raising awareness of SDM through public campaigns may help prepare individuals seeking health care to ask for SDM during consultations and subsequently increase uptake.^76^ HCPs should actively encourage predialysis individuals with kidney failure to participate in SDM to ensure that treatment decisions are not made without the participation of the individual and/or their family/caregiver.

### SDM in Emergency Situations

SDM may not be possible or may be limited in scenarios where urgent or unplanned kidney replacement therapy is required. Urgent kidney replacement therapy refers to dialysis initiation required within 48 hours of presentation, and unplanned dialysis is when the individual with kidney failure may require hospitalization to begin dialysis or when the modality is not their choice. HD and PD are options in most cases of urgent and unplanned dialysis; however, if the situation is critical, HCPs may not have the time or information to provide an SDM process. It is important that the individual with kidney failure is subsequently provided with the support and information required to transfer to the modality of their choice at a later date, if feasible.^77^

### Environmental Barriers to Uptake of SDM and PDAs

Environmental barriers to SDM may include lack of access to mobile technology or e-health. Another consideration is that older individuals seeking health care may not readily engage with technology and may not have ready access to or understand the complex use of technology in health care.^78^

Language, educational level, health literacy, and cultural barriers can prevent predialysis individuals with kidney failure from engaging in the SDM process.^61^ Older individuals with kidney failure are likelier to have poorer e-Health literacy, which may preclude their access to certain resources. Those from culturally or linguistically diverse backgrounds may be disadvantaged during SDM because of a lack of sufficient or understandable information and HCP time pressures.^79^ Consequently, they may miss out on opportunities to be an informed participant during the process. PDAs should be available in local languages and provided at an appropriate reading level. Individuals seeking health care can be influenced by many information sources that are readily accessible, and this can limit the degree to which they accept evidence-based knowledge provided by their HCP. Family members or other treatment-experienced individuals may influence new individuals with kidney failure, regardless of their level of knowledge, especially older adults who may lack autonomy on decision-making for their health.^61^ Indeed, filial piety, the honor and respect shown to parents, grandparents, and elderly relatives by children, is very influential in some cultures, particularly in Asia.^80^ Within these cultures, individuals may not be responsible for making their own treatment decisions, relying instead on family members to make decisions regarding their health care.

Learning styles vary between adults, with some preferring visual, auditory, or kinesthetic tools.^81^ This means that the delivery of SDM and PDAs should be adapted to meet the needs of the individual, without compromising the integrity of the material.

### The COVID-19 Pandemic

The COVID-19 pandemic poses barriers for continuing health care delivery, including SDM, with limitations on physical contact/proximity between HCPs and individuals with kidney failure. Challenges exist for predialysis individuals with kidney failure making decisions on starting dialysis and which options are most suitable.^82^ SDM meetings may have to be remote and the materials provided need to be available in a format suitable to be accessed from home-based settings. Of concern is that predialysis individuals may become more reliant on HCPs to advise them on treatment options.^82^ Predialysis individuals with kidney failure may need to be given more time to read/look at materials, as the social interactions and dynamics of virtual or telephone meetings differ from those during face-to-face consultations. This in turn may result in additional burden on the individual with kidney failure and the HCP in terms of time and resources. Use of COVID-19–specific decision aids may help initiate the dialogue needed for SDM in these circumstances.^82^ HCP training and support for SDM tailored to the COVID-19 era will be critical to ensure decision aids are used appropriately.^82^ Promoting home-based therapies may be beneficial in mitigating the challenges of in-center attendance (including possible transmission of coronavirus to individuals with kidney failure) posed by the COVID-19 pandemic, reducing the burden on dialysis care centers and prioritizing access to individuals who need it most. Similarly, those currently receiving ICHD may be encouraged to transition to HHD or PD where this is an option to reduce the impact on ICHD units and promote social distancing.^82^ It is important that risks and benefits to the individual be taken into consideration before suggesting these options.

### Lack of High-Quality Evidence

There is a lack of high-quality evidence regarding the effectiveness of interventions focusing on increasing the use of SDM among HCPs. A Cochrane systematic review recommended that future studies of SDM should be designed to minimize bias and methods and results should be fully reported. It highlighted that further research is needed to develop better patient-derived measures of SDM. Studies should be conducted across multiple clinical backgrounds and cost-effectiveness should be reported.^83^

There is also a lack of data regarding outcomes of SDM on dialysis modality choice, apart from the previously discussed positive impact on mental health. The impact of SDM on clinical outcomes, such as survival, is important for policy makers who may not see the utility of SDM without this information.

The barriers to SDM and proposed solutions are summarized in Table 2.

**Table 2:** Barriers to SDM and proposed solutions

Barrier to SDM	Solutions
Emotional burden experienced by individuals with kidney failure	
• Predialysis individuals may be fearful when choosing a dialysis modality	• Develop interventions that improve feelings of hope
Psychosocial factors and willingness to engage in SDM	
• Certain individuals, such as males or ethnic minorities, may be less willing to engage in SDM than other predialysis individuals • Emotional, psychosocial, and cognitive states can affect on treatment choice	• Family and community can help with informal education of the predialysis individual and identification of factors that influence engagement • Consider the psychological state when engaging in SDM
Lack of HCP engagement	
• HCPs have reported a lack of time and training to perform SDM • HCPs are unwilling to initiate SDM if it is not initiated by the predialysis individual	• Educate HCPs on the benefits of SDM • Encourage predialysis individuals to participate in SDM
SDM in emergency situations	
• SDM may not be possible when unplanned KRT is required	• Individuals who had unplanned KRT should be provided with support and information after the emergency situation has resolved (if feasible)
Environmental barriers to uptake of SDM and PDAs	
• Individuals with kidney failure may not be able to access mobile technology or e-health initiatives	• Develop PDAs to meet the needs of individual patients
COVID-19 pandemic	
• SDM may be affected by limitations on in-person meetings	• Improve access to virtual consultations
Lack of high-quality evidence	
• Lack of data on clinical outcomes with SDM	• Design studies that encompass a range of clinical scenarios • Report cost findings

HCP, health care provider; KRT, kidney replacement therapy; PDA, patient decision aid; SDM, shared decision-making.

### SDM Clinical Trials for Predialysis Individuals With Kidney Failure

There are several trials currently exploring SDM for predialysis individuals that will provide further guidance on the use of SDM.

#### *PREPARE NOW*

PREPARE NOW (NCT02722382) is a cluster-randomized controlled trial in the United States. The “Patient Centered Kidney Transition Care” program uses multicomponent interventions to improve patient preparedness for kidney replacement therapy through helping providers focus on patient values and treatment preferences. A primary outcome of the study is the change in the proportion of patients feeling in control of their decision-making at 36 months of follow-up. The study was completed in October 2020 and enrolled 1572 patients.^84^^,^^85^

#### *CKD-EDU*

The Feasibility of Enhanced Dialysis Education Intervention for Chronic Kidney Disease Patients (CKD-EDU) trial (NCT03465449) is a randomized, parallel controlled trial comparing palliative care-based dialysis decision-making with usual care. Patients will be ≥75 years of age and have CKD (stage 4/5). Primary outcomes are the number of patients receiving the intervention and the acceptability of the CKD-EDU. Estimated enrollment is 60 patients, and estimated completion is January 2022.^86^

#### *DIAL-SDM*

The Shared Decision Making in Dialysis (DIAL-SDM) trial is a randomized, single-blind pilot study (NCT04392440) planning to recruit patients aged ≥65 years with kidney failure/CKD who are facing challenges in decision-making on dialysis. The study will compare the DIAL-SDM intervention with usual care. Nephrologists in the intervention group will receive 3 communication training sessions, delivered by a standardized patient instructor. Patients (and caregivers, if available) will receive 2 coaching sessions provided by health coaches, who will explore each patient’s relevant contextual information (values, preferences, and goals) and help them identify and practice important questions for their nephrologist. Estimated enrollment is 60 participants, and estimated study completion is May 2023.^87^

### Summary and Recommendations

Access to SDM is a right that can lead to improved outcomes and can be integrated into the choice of treatment modality for predialysis individuals with kidney failure. SDM can empower people faced with decisions on which dialysis modality to choose and can lead to increased treatment satisfaction. It is immensely important that the perspective of “no decision about me, without me” be respected by HCPs when discussing their patients’ health care. For SDM to be successful, individuals seeking health care must be aware that the process is both active and iterative and requires their participation. Individuals with kidney failure must be equal partners if their long-term care is to be successful, and this partnership should begin at the very start of their dialysis journey. Within this partnership, HCPs should be mindful of the influences that may affect their patients’ decisions when choosing a treatment option. These can include personal values and preferences, age, or emotional state.

PDAs are integral to the SDM process. Although PDAs have been successfully integrated into kidney care in some health care systems, it is imperative that these tools are objective, evidence based, and up to date.

We recommend that all individuals with kidney failure are encouraged to participate in SDM to discuss their treatment options and should be involved in the design and implementation of recommendations regarding the SDM approach for those with CKD/kidney failure. This can be achieved by the formation of groups who are actively involved in providing input on SDM processes when choosing dialysis modality. Of course, some individuals will not want to participate in SDM and would prefer their HCPs make all their treatment choices. In this case, the individuals’ wishes should be respected.

Table 3 reveals our recommendations for a predialysis modality SDM consultation process between those seeking health care and HCPs, based on the 3-talk SDM model found in Figure 1. Although Table 3 describes our recommendations for predialysis individuals, it could be applied at any point on the dialysis journey as some individuals receive dialysis education only after commencing treatment (e.g., those who start dialysis in an emergency). It should also be noted that this process is dynamic, with changes in an individual’s medical condition or personal circumstances meaning that the process may need to be repeated with possible changes to previously made decisions.

**Table 3:** Recommendations for a predialysis modality SDM consultation process

Recommendations
Choice talk • Predialysis individuals with kidney failure should be invited to participate in an SDM choice talk after deciding to initiate dialysis: other options, such as conservative care, should have been excluded (ideally by using SDM) • HCPs should ask their patients to consider the values that matter most to them in regard to treatment (e.g., lifestyle choices) • The HCP should make their patient aware of the types of dialysis that are available. This will vary depending on region, but in general, the HCP will inform them of home- (CAPD, APD, or home HD) and hospital-based options (ICHD) • The consultation should take the form most suitable to the individual seeking health care; e.g., some may prefer a face-to-face meeting whereas others are happy with a virtual meeting • Predialysis individuals may start to consider their initial preference on dialysis modality, which can be discussed at the choice talk
Option talk • After the choice talk, a PDA should be provided to the individual with kidney failure to help with deliberation • The PDA should include all the key criteria described in the IPDAS Collaboration certification tool. Key criteria include the following: ○ Content: Information on different dialysis modalities, probability of outcomes (such as survival) with each dialysis modality, clarification of values suitable to the predialysis individual (e.g., if they prefer to have dialysis at home or at hospital), and disease-specific guidance regarding kidney failure ○ Development process: How the PDA was designed and developed, with specific reference to how the process was developed for individuals with kidney failure on to receive dialysis, planned updates, and funding source ○ Effectiveness: Evidence of the high-quality decision processes and results from trials comparing dialysis choice with and without the PDA • The PDA should be discussed at the option talk and any issues with usability identified • The predialysis individual should discuss any aspects of the PDA that they are struggling to understand • This talk should occur after the predialysis individual has had a suitable time period to review and use the PDA
Decision talk • The HCP and individual with kidney failure should review the talks held so far and identify if any of the predialysis individual’s circumstances or values have changed • Both the HCP and predialysis individual review the SDM process so far and identify any potential issues with the process (e.g., the predialysis individual is uneasy with the HCP’s influence on one aspect of their decision) • The individual with kidney failure should be prepared to make their informed final decision on dialysis modality • The HCP should provide their full support on the predialysis individual’s decision and initiate steps so treatment can commence

APD, automated peritoneal dialysis; CAPD, continuous ambulatory peritoneal dialysis; HCP, health care professional; HD, hemodialysis; ICHD, in-center hemodialysis; IPDAS, International Patient Decision Aids Standards; PDA, patient decision aid; SDM, shared decision-making.

It is incumbent on us all to ensure nephrology teams are trained in both SDM and the tools used to support this process.

## Disclosure

XY reports receiving grants from Baxter Healthcare Corporation, Kyowa-Kirin, China, Wanbang Company, AstraZeneca, and GlaxoSmithKline and providing consultancy for Baxter Healthcare Corporation. YLK reports receiving personal fees for speaker honorarium from Fibrogen. DS reports that Baxter is a sponsor of the nonprofit Medical Education Institute’s Home Dialysis Central website each year. RRQ reports receiving speaker fees from Baxter Corporation and having a patent “Dialysis Measurement Analysis and Reporting System” issued. MD is a full-time employee of Baxter Healthcare Pty Ltd. (Associate Medical Director, Medical Affairs—Baxter Healthcare ANZ). All the other authors declared no competing interests.
